# Trait-mediated shifts and climate velocity decouple an endothermic marine predator and its ectothermic prey

**DOI:** 10.1038/s41598-021-97318-z

**Published:** 2021-09-16

**Authors:** L. H. Thorne, J. A. Nye

**Affiliations:** 1 School of Marine and Atmospheric Sciences, Stony Brook University, Stony Brook, NY 11794-5000 USA; 2 Institute of Marine Sciences, University of North Carolina Chapel Hill, Chapel Hill, NC USA

**Keywords:** Food webs, Climate-change ecology, Biogeography, Animal physiology, Marine biology, Ecology, Ocean sciences

## Abstract

Climate change is redistributing biodiversity globally and distributional shifts have been found to follow local climate velocities. It is largely assumed that marine endotherms such as cetaceans might shift more slowly than ectotherms in response to warming and would primarily follow changes in prey, but distributional shifts in cetaceans are difficult to quantify. Here we use data from fisheries bycatch and strandings to examine changes in the distribution of long-finned pilot whales (*Globicephala melas*), and assess shifts in pilot whales and their prey relative to climate velocity in a rapidly warming region of the Northwest Atlantic. We found a poleward shift in pilot whale distribution that exceeded climate velocity and occurred at more than three times the rate of fish and invertebrate prey species. Fish and invertebrates shifted at rates equal to or slower than expected based on climate velocity, with more slowly shifting species moving to deeper waters. We suggest that traits such as mobility, diet specialization, and thermoregulatory strategy are central to understanding and anticipating range shifts. Our findings highlight the potential for trait-mediated climate shifts to decouple relationships between endothermic cetaceans and their ectothermic prey, which has important implications for marine food web dynamics and ecosystem stability.

## Introduction

While shifts in distribution^1–4^ and productivity^5,6^ in response to warming have been well documented in marine ectotherms^7,8^, direct impacts of climatic warming on marine endotherms are poorly understood^9^. As a result of differences in thermoregulatory mechanisms and energetic expenditure when faced with changing environmental temperatures, climate change may have fundamentally different impacts on ectotherms and endotherms^10^. It is largely assumed that ectotherms will respond more quickly to warming than endotherms^11,12^. The physiological performance of marine ectotherms is a function of water temperature and thus warming has had marked and measurable impacts on their growth, population productivity, and spatial distribution^13–15^. In contrast, endotherms have a greater capacity to maintain a constant body temperature by altering metabolic heat production in response to temperature fluctuations, but physiological thermal limits still constrain spatial distribution at broad scales^16^.

Understanding the pace of climate change, or climate velocity, is key to understanding species responses in a given habitat^17,18^. It is generally thought that species must keep up with the pace of climate change in order to survive^4,17^. However, there is some controversy as to the relative degree to which climate velocity versus species traits explain range shifts. In marine ecosystems, previous studies have found that species traits do not strongly influence a species’ response to climate velocity^4,19^ while others have found that species’ traits were key to explaining range shifts^20^. However, these studies focus on ectotherms. For endothermic marine predators, the relative importance of direct thermoregulatory responses vs. indirect effects mediated by prey dynamics in marine predators is critical to understanding how they may respond to climate change^21,22^. Since marine mammals are large and abundant endothermic predators with higher metabolic rates than ectotherms, their large prey requirements regulate marine foodwebs^23–25^ and differential climate responses between these predators and their ectothermic prey would have formidable impacts on the resilience and stability of marine ecosystems^26,27^. Thus, considering traits including thermoregulatory strategy is critical to understanding species range shifts across many taxa.

For endotherms, the use of elevated metabolism to maintain a constant body temperature consumes a major part of the energetic budget at environmental temperatures above or below the thermoneutral zone^28^. Cetaceans spend their whole life underwater, and thus warming waters may be more likely to directly impact thermoregulation in cetaceans than other marine endotherms such as flying seabirds that are not constantly immersed in a highly conductive environment. While thermal tolerances of cetaceans are not well understood, in broad terms, different taxonomic groups of cetaceans are known to vary considerably in their thermal tolerances. Baleen whales undertake long annual migrations between high latitude feeding grounds and tropical breeding grounds and are adapted to occur in a wide range of temperatures^29^. Conversely, most odontocetes have more limited ranges and their morphology and physiology reflect adaptations to the thermal conditions in their range. For example, cooler water species typically have lower surface area to volume ratios in their body core, and blubber with a higher lipid content, lower conductivity, and increased fatty acid stratification^30,31^. The larger appendages of long-finned pilot whales in comparison to short-finned pilot whales may reflect the need for enhanced mechanisms of heat dissipation in the large, well-insulated long-finned species, which occurs in cooler waters^32^.

Distributions of many cetacean species are expected to move poleward under climate change^33,34^, resulting in range contractions for cool water species, poleward distributional shifts for temperate species, and range expansions for warm water species^21^. There is evidence that considerable distributional shifts have occurred in marine mammal species in recent decades, with changes in the occurrence of cool and warm water cetacean species occurring in association with warming waters^35–38^. However, our ability to quantify distributional shifts in marine mammals is limited by the lack of reliable time series of abundance and distribution for most species^36^.

Measuring long-term changes in cetacean populations presents a considerable challenge for biologists and managers^39,40^; at-sea sightings rates from line transect surveys used to assess abundance are low, necessitating high survey effort to produce abundance estimates^41^. Consistent, standardized surveys with sufficient temporal and spatial coverage to detect distributional shifts over decades are not typically feasible and standardized monitoring effort for cetaceans in US waters is likely insufficient for detecting trends in the abundance and distribution of cetacean populations^40,41^. Observations from stranding events and fisheries bycatch provide important means of revealing changes in cetacean distribution over time, and may sample cetacean diversity better than surveys of live animals^37,39,42^. Further, some marine mammal species are difficult to identify at sea, and strandings records and observations of fisheries bycatch by onboard observers allow species-specific trends to be examined for species which are otherwise difficult to identify.

Here, we examine changes in the distribution of the long-finned pilot whales (*Globicephala melas*) relative to their fish and invertebrate prey over a 25-year period in the Northeast United States (NEUS). We use strandings and bycatch data to examine changes in the distribution of long-finned pilot whales, and analyze catch data from fisheries-independent trawl data to examine long-term trends in the distribution of four dominant prey species of long-finned pilot whales, long-finned squid (*Doryteuthis pealeii*), short-finned squid (*Illex illecebrosus*), Atlantic mackerel (*Scomber scombrus*), and Atlantic herring (*Clupea harengus*). We test the hypothesis that both predator and prey, which differ considerably in their species traits, will shift at the same rate relative to local climate velocity. We also examine rates of fisheries bycatch relative to SST for evidence of thermal limits to pilot whale distribution and test the assumption that the endothermic predator will shift more slowly or lag their ectothermic prey.

## Results

**Figure 1:**
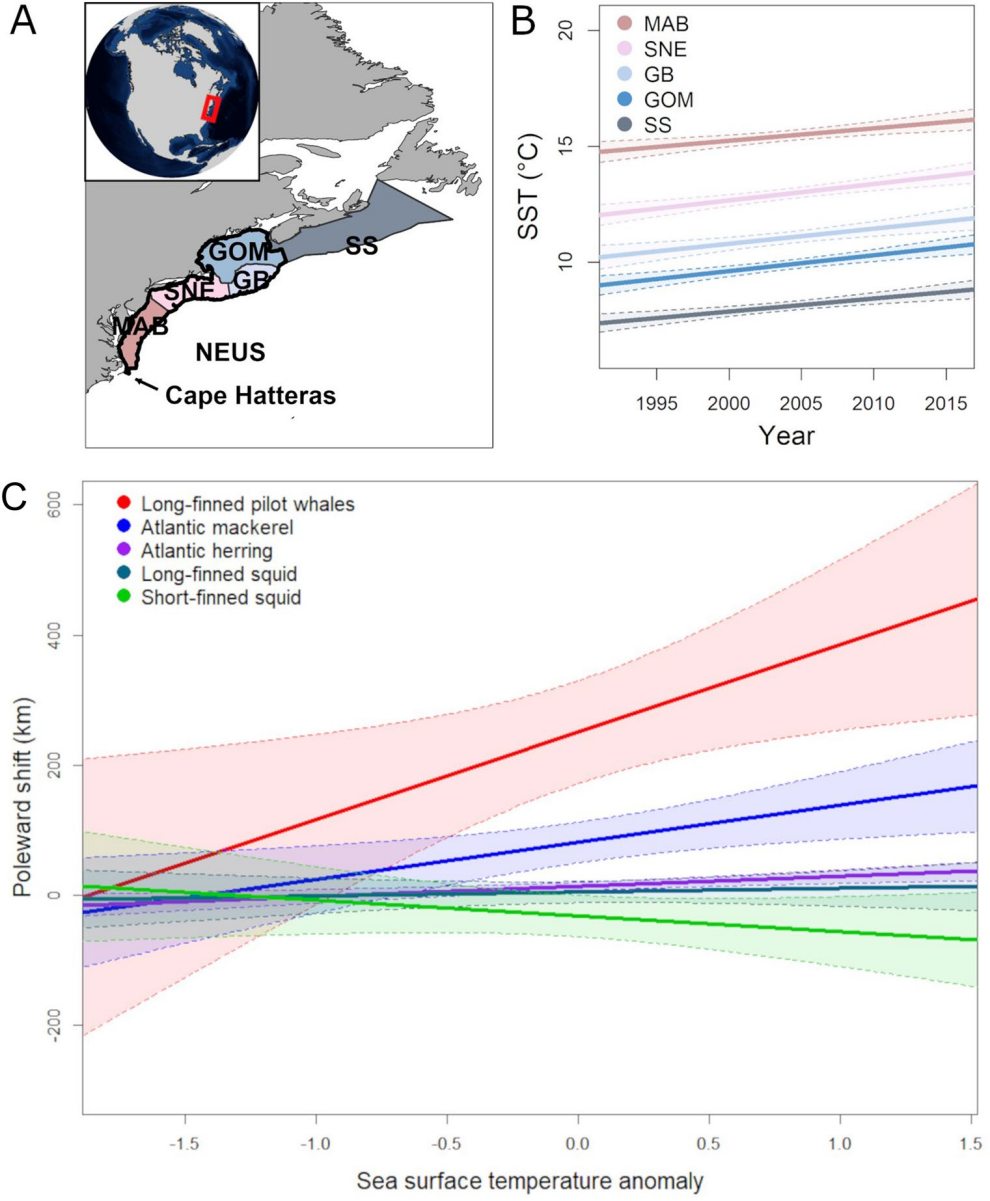
Waters in the Northeast United States (**A**) have warmed rapidly in recent decades (**B**, mean fall sea surface temperature). A poleward shift in a marine endotherm (long-finned pilot whales) was greater and more strongly linked with sea surface temperature (SST) than that of their ectothermic prey species (Atlantic mackerel, Atlantic herring, longfin squid and shortfin squid; **C**). Poleward shifts were calculated relative to the beginning of the study period while sea surface temperature anomalies were calculated during the 1992–2016 study period. MAB = Mid-Atlantic Bight; SNE = Southern New England; GB = Georges Banks; GOM = Gulf of Maine; SS = Scotian Shelf. Map produced using ArcGIS (v. 10.8.1).

**Figure 2:**
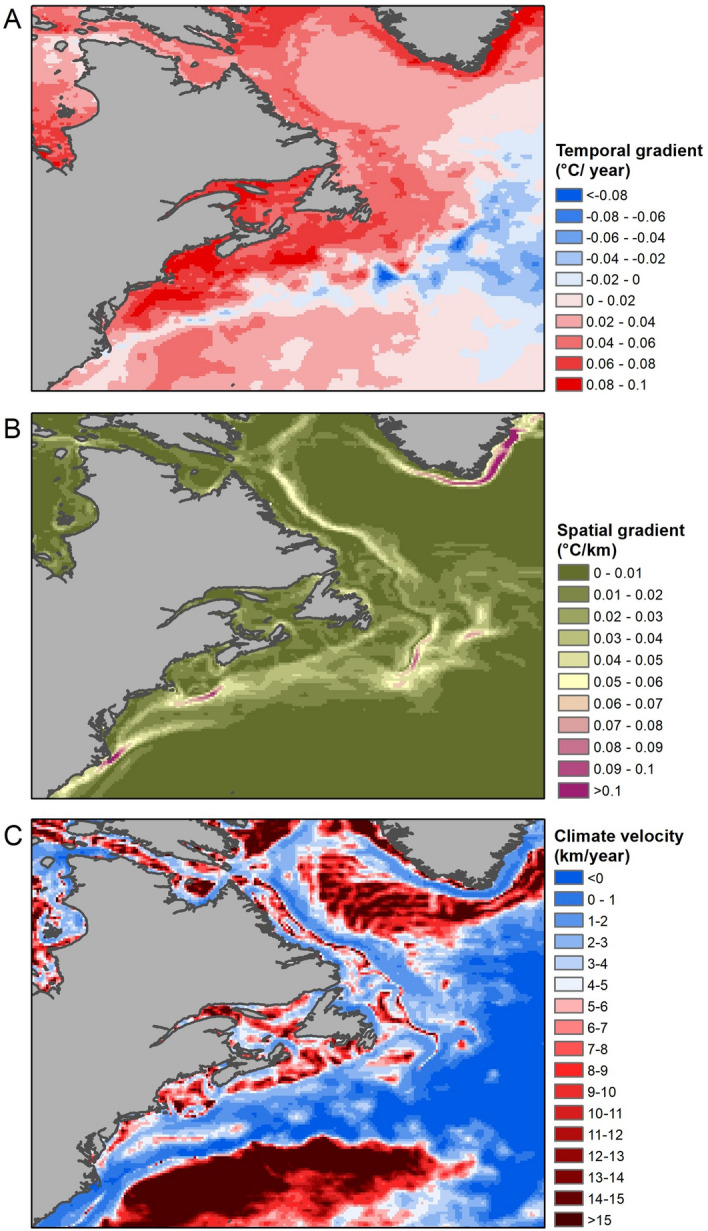
Temporal (**A**) and spatial (**B**) gradients in fall sea surface temperature used to calculate the velocity of climate change (**C**) in the Northwest Atlantic, calculated from 1992–2016. Climate velocity represents the rate at which isotherms move. Higher values of climate velocity represent faster poleward movement of isotherms. Maps produced using ArcGIS (v. 10.8.1).

**Figure 3:**
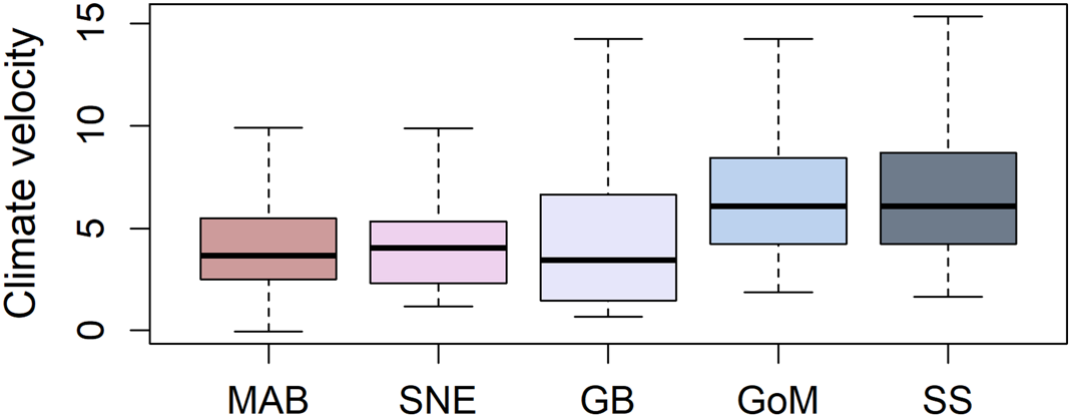
Median climate velocity (km/year) within the Mid-Atlantic Bight (MAB), Southern New England (SNE), Georges Bank (GB) and Gulf of Maine (GOM) subregions of the Northeast US, as well as the Scotian Shelf (SS) in Canada.

From 1992 to 2016, mean fall SST in long-finned pilot whale habitat in the NEUS increased linearly through time (Adjusted R^2^ = 0.47, p = 9.55 × 10^–7^, slope = 0.074 °C year^−1^; Table 1, Fig. 1). Temporal trends in SST were generally consistent between subregions in the NEUS (Fig. 1B), though the Gulf of Maine, Georges Bank and Southern New England regions showed particularly high mean rates of change, at between 0.65 and 0.70 °C year^−1^ (Table 1, Fig. 2A). Spatial gradients in SST were lowest in the Gulf of Maine and Scotian Shelf regions (Fig. 2B). Together these spatial and temporal gradients resulted in higher climate velocities in the Gulf of Maine and Scotian Shelf (Fig. 2C, Fig. 3).

**Table 1:** Estimated parameters of linear regressions used to evaluate changes in the poleward distance and depth of the center of distribution for long-finned pilot whales, Atlantic mackerel, Atlantic herring, longfin squid and shortfin squid from 1992 to 2016. Significant relationships (p < 0.05) are shown in bold. MAB = Mid-Atlantic Bight; SNE = Southern New England; GB = Georges Banks; GOM = Gulf of Maine; SS = Scotian Shelf.

Model	Estimate	Standard error	t value	Pr(> t)	Adjusted R^2^
**Temperature models**					
Sea surface temperature NEUS ~ year	**7.37E−02**	**1.56E−02**	**4.72**	**9.46E−05**	**0.47**
Sea surface temperature SS ~ year	**5.60E−02**	**1.29E−02**	4.34	**2.40E−04**	**4.3E−01**
Sea surface temperature GOM ~ year	**6.82E−02**	**1.35E−02**	**5.04**	**4.21E−05**	**0.50**
Sea surface temperature GB ~ year	**6.49E−02**	**1.67E−02**	**3.88**	**7.55E−04**	**0.37**
Sea surface temperature SNE ~ year	**7.03E−02**	**1.47E−02**	**4.78**	**8.04E−05**	**0.48**
Sea surface temperature MAB ~ year	**5.40E−02**	**1.47E−02**	**3.68**	**1.24E−03**	**0.34**
**Pilot whale models**					
Poleward distance ~ year	**22.53**	**3.67**	**6.14**	**2.89E−06**	**0.60**
Poleward distance ~ sea surface temperature	**121.66**	**54.43**	**2.24**	**3.59E−02**	**0.15**
**Atlantic mackerel models, 1980–2019**					
Poleward distance ~ year	**4.09**	**1.13**	**3.62**	**8.59E−04**	**0.24**
Depth ~ Year	4.96E−02	0.15	0.33	0.75	− 2.5E−02
**Atlantic mackerel models, 1992–2016**					
Poleward distance ~ year	**7.13**	**1.94**	**3.68**	**1.24E−03**	**0.34**
Depth ~ Year	1.05	0.54	1.94	6.42E−02	0.10
Poleward distance ~ sea surface temperature	**64.09**	**20.55**	**3.12**	**4.82E−03**	**0.27**
**Atlantic herring models, 1980–2019**					
Poleward distance ~ year	**1.94**	**0.30**	**6.41**	**1.73E−07**	**0.51**
Depth ~ Year	**1.62**	**0.21**	**7.90**	**1.86E−09**	**0.62**
**Atlantic herring models, 1992–2016**					
Poleward distance ~ year	**1.62**	**0.42**	**3.84**	**8.40E−04**	**0.36**
Depth ~ year	**0.99**	**0.26**	**3.82**	**8.85E−04**	**0.36**
Poleward distance ~ sea surface temperature	**17.77**	**4.41**	**4.03**	**5.21E−04**	**0.39**
**Longfin squid models, 1980–2019**					
Poleward distance ~ year	5.00E−02	0.56	8.9E−02	0.93	− 2.7E−02
Depth ~ year	**0.31**	**0.10**	**3.10**	**3.67E−03**	**0.18**
**Longfin squid models, 1992–2016**					
Poleward distance ~ year	− 1.61	1.06	− 1.52	0.14	5.2E−02
Depth ~ year	**0.88**	**0.17**	**5.27**	**2.42E−05**	**0.53**
Poleward distance ~ sea surface temperature	9.09	9.73	0.93	0.36	− 5.4E−03
**Shortfin squid models, 1980–2019**					
Poleward distance ~ year	− **2.68**	**1.15**	− **2.32**	**2.59E−02**	**0.11**
Depth ~ year	**0.96**	**0.24**	**4.06**	**2.36E−04**	**0.28**
**Shortfin squid models, 1992–2016**					
Poleward distance ~ year	− 2.49	2.11	− 1.18	0.25	1.6E−02
Depth ~ year	**2.48**	**0.36**	**6.85**	**5.48E−07**	**0.66**
Poleward distance ~ sea surface temperature	− 17.88	17.24	− 1.04	0.31	3.3E−03

**Figure 4:**
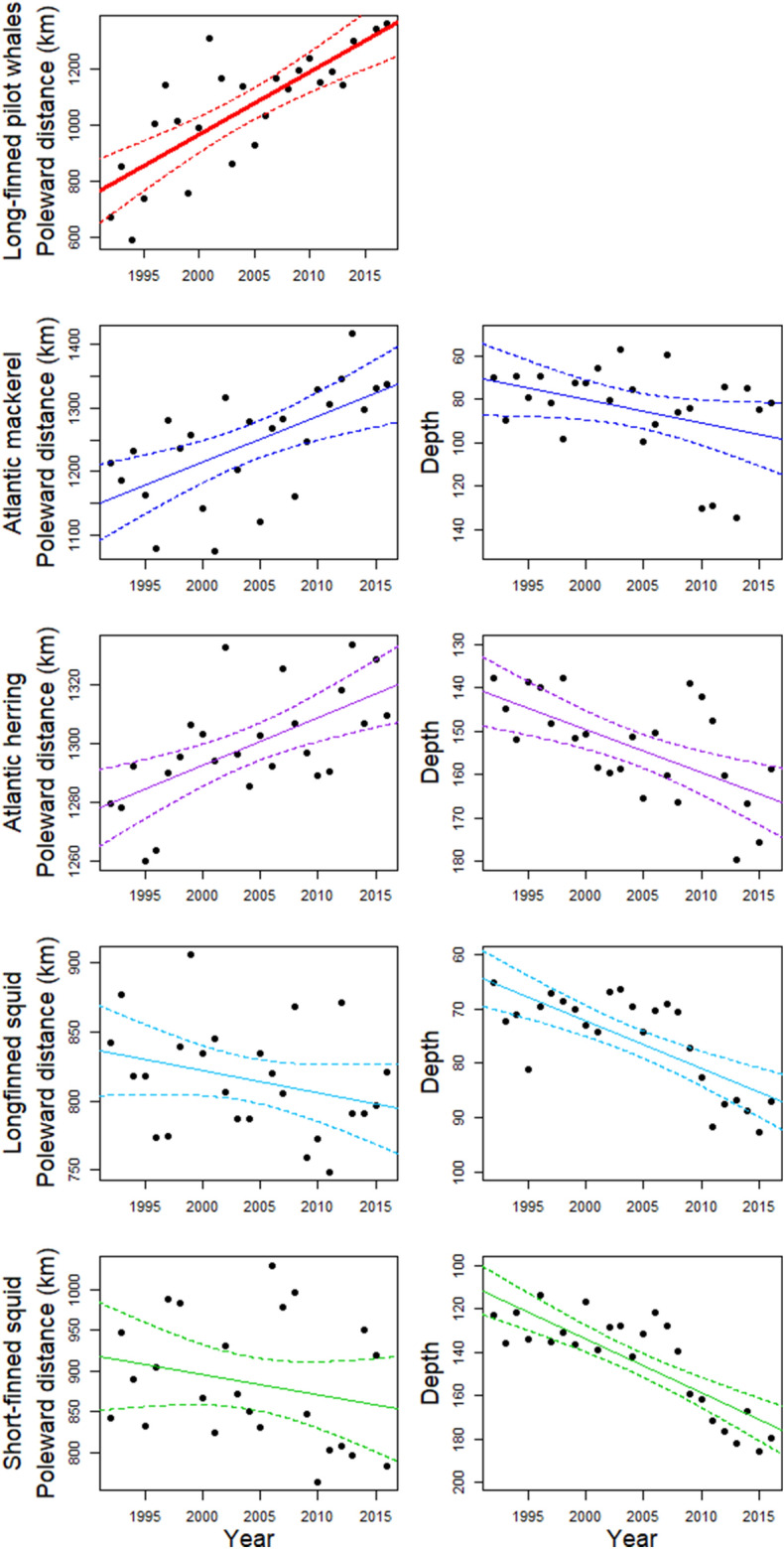
Changes in the mean poleward distance (left panels) and depth (right panels) in the center of distribution of study species from 1992 to 2016. Poleward distance reflects the along-shelf distance measured from Cape Hatteras, North Carolina.

**Figure 5:**
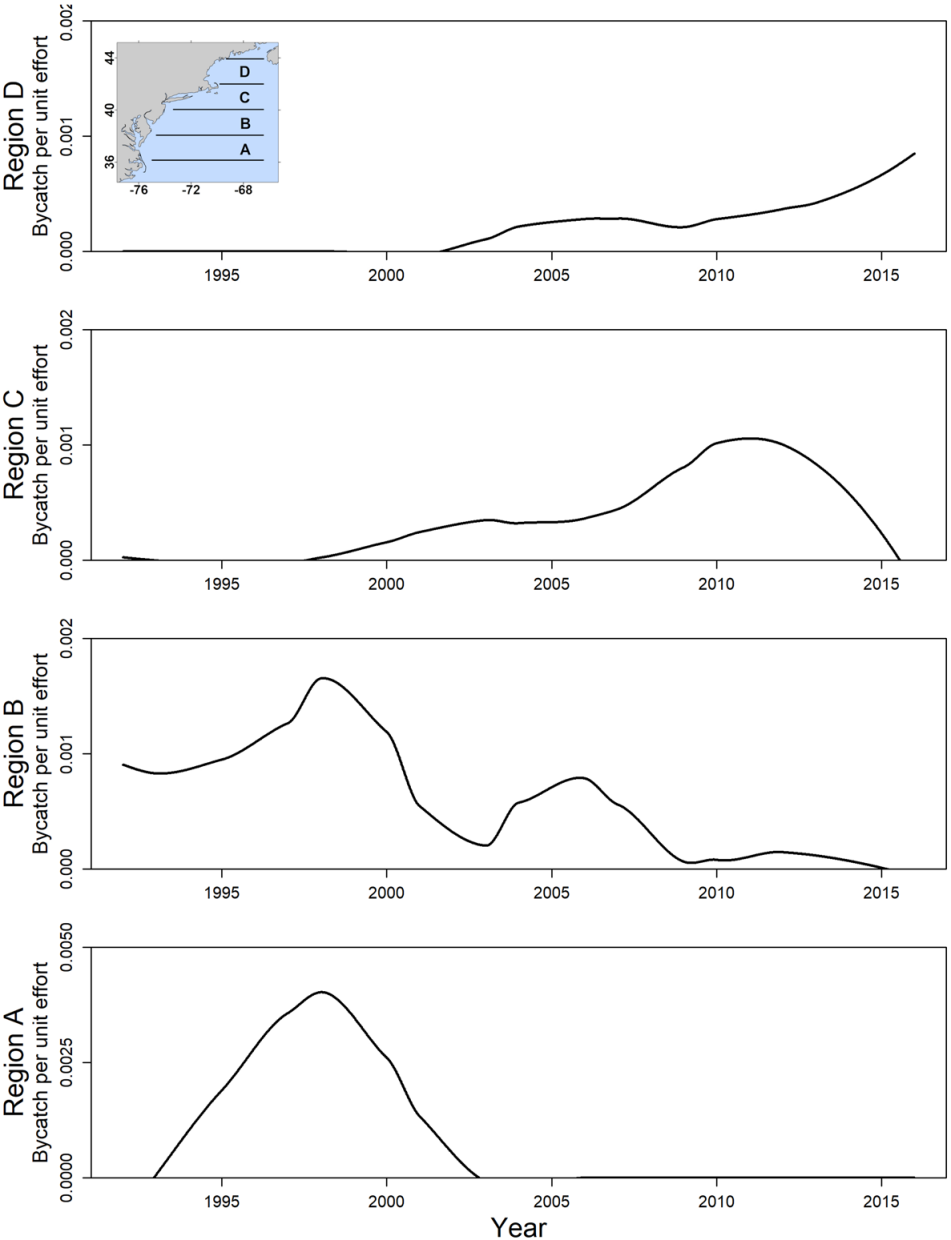
Within the Northeast United States, long-finned pilot whale bycatch shifted north from 1992 to 2016. Pilot whale bycatch per unit effort for bottom trawls through time is shown within four latitudinal bands in the Northeast US. The location of the bands is shown in the inset.

The mean poleward distance of pilot whale observations increased linearly through time (Adjusted R^2^ = 0.60, p = 2.89 × 10^–6^, slope = 22.5 km year^−1^; Fig. 4) and increased linearly with SST (Adjusted R^2^ = 0.15, p = 3.6 × 10^–2^; Fig. 1C; Table 1). There were no corresponding shifts in the spatial distribution of bottom trawls that could explain shifts in bycatch data (Supplementary Figs. 1–2). Further, when corrected for effort, pilot whale bycatch per unit effort (BPUE) in the bottom trawl fishery shifted poleward through time, with higher BPUE occurring at high latitudes further north in later years (Fig. 5, Supplementary Fig. 3). Rates of pilot whale BPUE were lower in later years (Fig. 5, Supplementary Fig. 3), suggesting that in recent years the distribution of long-finned pilot whales might have shifted so far north that the species was observed less frequently within the NEUS. While trawls frequently occurred above 20 °C, pilot whale bycatch rarely occurred above this temperature (Supplementary Fig. 4), suggesting a thermal limit in the realized niche of this species. Bottom trawls occurred in warmer waters in later years (linear regression, Adjusted R^2^ = 0.31, p = 2.1 × 10^–3^), illustrating that while the spatial distribution of bottom trawls did not change, the temperature increased in the region where bottom trawls were conducted.

Temporal trends in the distribution of two of the four pilot whale prey species showed no poleward shifts over the 25-year study period, while two species, Atlantic mackerel (*Scomber scombrus*) and Atlantic herring (*Clupea harengus*), showed a significant poleward shift in distribution (Fig. 4, Table 1, Supplementary Figs. 5–8; for mackerel, Adjusted R^2^ = 0.34, p = 1.2 × 10^–3^, slope = 7.1 km year^−1^; for herring, Adjusted R^2^ = 0.36, p = 8.4 × 10^–4^, slope = 1.6 km year^−1^). Atlantic herring, longfin squid (*Doryteuthis pealeii*) and shortfin squid (*Illex illecebrosus*) showed significant shifts into deeper waters (Fig. 4, Table 1; for herring, Adjusted R^2^ = 0.36, p = 8.8 × 10^–4^, slope = 0.99 m depth year^−1^; for longfin squid, Adjusted R^2^ = 0.53, p = 2.4 × 10^–5^, slope = 0.88 m depth year^−1^; for shortfin squid, Adjusted R^2^ = 0.66, p = 5.5 × 10^–7^, slope = 2.5 m depth year^−1^). Poleward shifts in Atlantic mackerel and Atlantic herring were associated with increasing SST, but long-finned pilot whales shifted poleward more than twice as rapidly as their prey in response to increasing SST (Fig. 1C, Table 1). The shift in prey center of biomass accelerated later in the time period, reflecting the more rapid increase in temperature since the early 2000s (Table 1), but still remained slower than the shift observed in pilot whales. Although no significant poleward shift in distribution was detected in either squid species, there was a significant shift to deeper depths.

**Figure 6:**
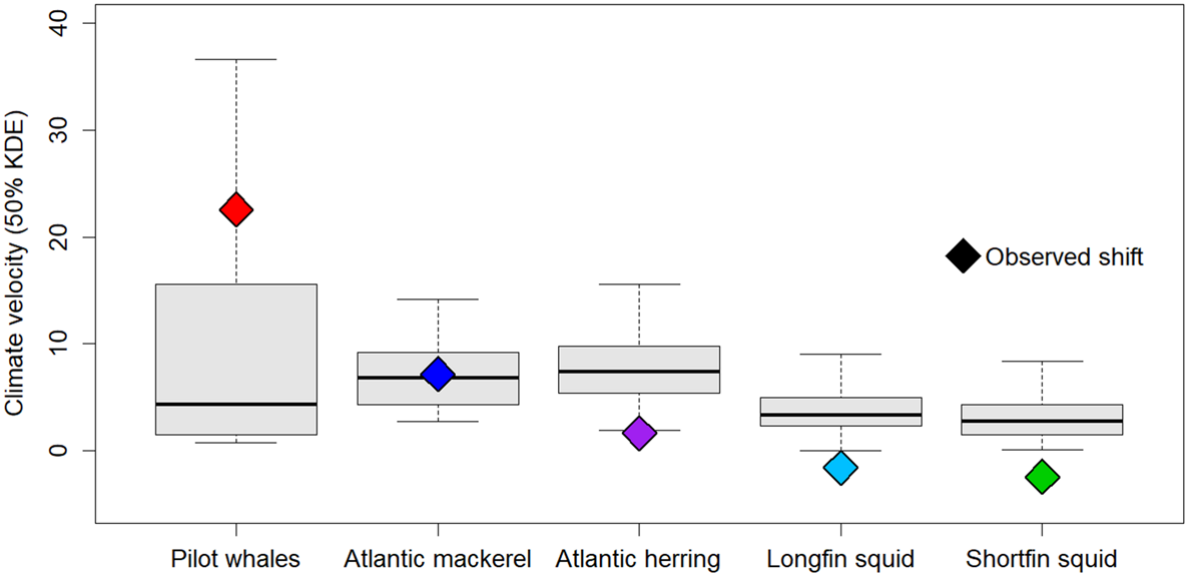
Climate velocity (km/year) within the core habitat of long-finned pilot whales, Atlantic mackerel, Atlantic herring, longfin squid and shortfin squid. Colored diamonds represent observed poleward shifts for each species. Pilot whales shifted faster than expected based on climate velocity in their habitat, while Atlantic mackerel shifted approximately as expected and Atlantic herring, longfin squid, and shortfin squid shifted less than expected.

The distribution of longfin and shortfin squid extended from the Mid-Atlantic Bight to Georges Bank, while that of Atlantic herring and Atlantic mackerel was focused in the Gulf of Maine and Georges Bank, where climate velocities were higher (Figs. 2C, 3). Long-finned pilot whales primarily occurred from Southern New England into Georges Bank and the Gulf of Maine. Calculations of climate velocity within species’ core habitats suggested that long-finned pilot whales, Atlantic mackerel and Atlantic herring would need to show greater poleward shifts than longfin and shortfin squid in order to keep up with the pace of climate change (Fig. 6). Observed distributional shifts were higher than expected for pilot whales, within the expected range for Atlantic mackerel, and lower than expected for Atlantic herring, longfin squid and shortfin squid (Fig. 6).

## Discussion

To our knowledge this is the first quantified poleward shift in distribution of an odontocete in response to rapidly warming surface waters. While field-based observations have indicated that range shifts in cetaceans are likely occurring^35,37,43^ and modeling studies highlight the potential for future climate-driven shifts in distribution^44^, we are not aware of studies that have quantified long-term spatial shifts in cetacean distribution to date. The observed poleward shift in long-finned pilot whale distribution is striking in that it represents poleward movement at a rate of approximately 22 km year^−1^at the southern limit of the species’ range in the Northwest Atlantic, a faster shift than has been observed in the center of biomass and trailing edge of 95% other marine species^45^. Whether such rates are common in other mobile marine mammals is critically needed to anticipate ecosystem effects. Our finding that the climate-induced geographic shifts of pilot whales exceed those of ectothermic prey suggests that pilot whales are showing a direct response to warming waters rather than following changing distributions in their prey.

Rather than lagging distributional shifts in their ectothermic prey, the observed rate of poleward movement in long-finned pilot whales is higher than shifts observed in the prey species observed in this study (poleward movements of 7 and 2 km year^−1^, respectively, for Atlantic mackerel and Atlantic herring) and higher than any rate reported for fishes and macroinvertebrates in this region^2,4^. The shifts in herring and mackerel in the last 25 years are greater than those assessed using data extending to the 1960s^2^ likely due to the rapid warming that occurred from 2003 to present^46^. Further, long-finned pilot whales shifted at a rate that was considerably higher than climate velocity within the NEUS, but fish and invertebrates shifted as expected or slower than expected based on climate velocity. Of the five study species, only 1 was found to be shifting at a rate reflective of climate velocity. Thus, we suggest that climate velocity alone is not sufficient for understanding the varied responses to warming in species that differ in key traits.

The rapid shift in pilot whales relative to their prey is consistent with the ecological theory of trait-mediated climate shifts. Large climate-driven shifts in generalist predators like pilot whales are supported by the idea that generalists and species with greater dispersal capabilities and larger latitudinal ranges should shift faster^20^. Long-finned pilot whales are large, highly mobile predators that are capable of moving thousands of kilometers (horizontally) in a matter of weeks^47,48^, and pilot whales do not have fixed breeding or feeding areas like some other marine mammals. The range of long-finned pilot whales in the Northwest Atlantic extends from the NEUS into the Labrador Sea, and higher rates of climate velocity were observed in more northerly waters of the Northwest Atlantic (Figs. 4, 5). While movements and stock structure of long-finned pilot whales are uncertain, long-finned pilot whales are thought to move into more northern waters in late spring^49^. The observed shift in pilot whale distribution may better reflect the pace of climate change within their habitat in waters north of the NEUS. Long-finned pilot whales have high interannual variability in diet suggesting that they are generalist predators that may alter their diet based on prey availability^50^. Thus, pilot whales can target different prey species if climate change alters their distribution relative to a prey species that was previously common in their diet. In contrast, fish and squid possess traits that are hypothesized to lead to comparatively slower range shifts, such as smaller body size and lower adult motility in comparison to pilot whales. While fish can undertake large seasonal migrations^51^, they have a lower capacity to move and explore the environment in comparison to cetaceans^52^. Some fish species may return annually to spawning grounds which ultimately constrains their distributional response to climate change.

The ability of fish and squid to move to cooler waters by moving deeper in the water column likely plays an important role in the observed mismatch in climate responses between pilot whales and their prey. Long-finned pilot whales are deep diving species that can dive to depths of more than 800 m^47,53,54^, but as air-breathing mammals, pilot whales are constrained in their ability to spend time at depth. Deep foraging dives of long-finned pilot whales are brief (< 18 min), and the vast majority of their time is spent in waters of less than 15 m depth; thus surface temperatures play a larger role in their thermal budget than might be expected for marine mammals that spend more time at depth^47,53,55^. In contrast, fish and invertebrates occur throughout the water column and can shift poleward, offshore and/or deeper in order to stay within their preferred temperature ranges^56,57^. Distributional shifts in fish and macroinvertebrates in this region are generally more strongly associated with bottom rather than surface temperatures^4,58,59^ and bottom temperatures are also warming at a slightly slower rate than surface waters in this region^60^. Our results suggest that these species may be compensating for warming by shifting to deeper depths rather than shifting to more northern waters. Similarly, Pinsky et al.^4^ found that in regions of increasing SST, fish assemblages tended to shift deeper to avoid warming waters. However, the bottom trawl data that we examined do not fully capture the distribution of prey species in the water column and the relative importance of poleward shifts and shifts in depth merit further attention.

The shift in distribution of marine fishes can also partially be attributed to changes in population productivity with populations at the northern range limit having higher population growth rates than those at the southern limits. While the pelagic prey that we examined have relatively fast generation times, changes in population productivity as a mechanism for poleward shifts in distribution may explain their slower pace of range extensions in comparison to highly mobile marine predators where the mechanism of distributional shifts is directed movement.

Physiological and ecological processes underlying latitudinal trends in species richness for endotherms and ectotherms may also play a role in the rapid distributional shift we observed in long-finned pilot whales. For ectothermic prey species, important biological processes such as swimming speed and metrics of visual abilities are slower in cooler waters. As a result, prey in cool waters are easier for endothermic mammals and birds to capture while predatory sharks are easier to avoid, and marine endotherms thus have a competitive advantage over ectothermic predators in cooler waters^61^. Endothermic predators, while capable of tolerating a wide range of temperatures, may have a competitive predatory advantage by shifting faster.

The role of thermoregulation in mediating climate responses of endothermic predators relative to their prey merits further investigation. Endotherms such as cetaceans are capable of tolerating a wide range of temperatures through both behavioral and physiological mechanisms^62,63^, but given the high energetic costs of endothermy outside of the thermoneutral zone^64^, these species might be expected to show rapid climate responses at the edge of their ranges should water temperatures fall outside of the thermoneutral zone. The upper and lower critical limits of the thermoneutral zone are unclear for most cetacean species since traditional study methods such as respirometry are typically not feasible (though see^65^). It is therefore not currently possible to assess habitat shifts in cetaceans relative to thermal tolerance. However, endothermy in the highly conductive marine environment is associated with a number of other traits, such as large body size and thus high motility, that are expected to influence climate responses^20^. Thus, whether due to direct effects of endothermy or associated traits, cetaceans may be expected to show climate responses that differ from their prey species. Of course, species-specific traits and factors such as fixed breeding areas, observed in baleen whales, may also influence climate responses of cetaceans.

The extent to which our findings apply to other cetacean species and ecosystems is unclear given the lack of reliable time series to examine the effects of environmental change on cetaceans^36^. Our results also suggest that the relative shift of cetaceans and their prey may depend on the extent to which surface temperatures influence the thermal budget of cetacean species, and the ability of fish and invertebrate prey species to move deeper in the water column rather than poleward to stay within their preferred temperature range. Further, the climate velocity observed in the NEUS exceeds that expected for other regions, including nearby regions such as the South Atlantic Bight, given the rapid pace of climate change observed in the northern reaches of the NEUS. Our estimates of climate velocity were calculated at a finer spatial scale than previous studies (0.2 degrees rather than 1 degree; e.g.,^18,57^) and thus resulted in greater spatial variability and a higher range of climate velocities than observed in previous studies. Analyzing SST at a higher resolution is particularly important in the NEUS and broader Northwest Atlantic in order to accurately resolve the position of the Gulf Stream and temperatures on the continental shelf^66^. This approach allowed us to examine differences in SST gradients and climate velocities between subregions and species habitats within the NEUS.

Our analyses of changes to the spatial distribution of pilot whales and their prey suggests that in recent years, long-finned pilot whale diet may have changed. Few studies have quantified the diet of long-finned pilot whales and thus our current understanding of long-finned pilot whale diet may be incomplete and may not reflect temporal changes in diet. We focus here on the best available data, taken from studies conducted in the early 1990s^50^, which suggest that longfin squid, shortfin squid, Atlantic mackerel and Atlantic herring dominate the diet of long-finned pilot whales. Long- and short-finned squids which were found to be prevalent in long-finned pilot whale diet in the NEUS in the early 1990s may represent a smaller contribution to pilot whale diet in this region today due to the marked shifts observed in pilot whale but not squid distribution. Contemporary studies of long-finned pilot whale diet in the NEUS would allow these predictions to be assessed.

The implications of mismatches between cetaceans and their prey are considerable. Interactions between predators and prey play a major role in maintaining the structure and stability of ecosystems^26,27^, and bioenergetics studies indicate that marine mammal predation on fish stocks in the NEUS is significant and may equal removal by commercial fisheries^67–70^. While large whales have higher daily consumption rates, the annual population-level consumption of smaller odontocetes can rival or exceed that of large whales due to their higher abundance and longer residence in the NEUS. For example, pilot whales are estimated to consume more than 60 thousand metric tons of squid each year, more than three times as much squid as any other marine mammal species and considerably more squid than is removed by commercial fisheries in the NEUS each year^70^. Our results suggest that long-finned pilot whales have shifted poleward and today occur less frequently in the southern reaches of the NEUS than they did previously. Declines in top predators and mismatches in the occurrence of predators and prey have the potential to disrupt trophic interactions and can restructure marine communities^71–76^. Thus, our findings highlight the importance of assessing how climate change is changing the distribution of cetaceans relative to their prey in order to understand the ecological implications of future climate-driven change.

We show here that shifts in the distribution of a cetacean species was correlated with thermal niches (SST) rather than shifts in the distribution of its prey. Our results suggest that species traits such as thermoregulatory strategy, motility, body size, depth tolerance, and diet specificity may dictate whether or not species track climate velocity. We found that range shifts of marine endotherms can outpace shifts in their ectothermic prey species because the mechanisms underlying the shifts differ for predator and prey. Climate-driven shifts of predators that are decoupled from those of their prey may have broad implications for food web ecology and ecosystem structure.

## Methods

### Study site

The Northeast United States (NEUS) is a highly productive marine ecosystem bounded by Cape Hatteras, NC to the south, the Scotian Shelf to the north and the Gulf Stream to the east that supports important commercial and recreational fisheries. The NEUS lies at the nexus of warm, salty slope water associated with the Gulf Stream moving north and cold, less-saline Labrador slope water flowing south. Water properties at depth in the NEUS are heavily influenced by the volume of equatorward-flowing Labrador Slope water such that when the volume of Labrador Slope water is high, water is cold and fresh on the shelf. When the volume of Labrador Slope water is low, the Gulf Stream is in a more northerly position and water on the shelf is warm and salty. As these two water masses converge in the NEUS, they create a strong gradient in temperature. In summer, the temperature gradient can differ by almost 20 °C from Cape Hatteras to Nova Scotia, but high interannual variability in water temperature causes the location of isotherms to vary greatly. Water temperatures have shown some of the highest rates of warming in the world in recent decades^46^, but detailed oceanographic assessments of climate velocity within the NEUS that are independent of species distribution data and account for both spatial and temporal gradients in temperature are lacking.

### Study species

Long-finned pilot whales are deep-diving odontocetes that feed on fish and squid and are capable of diving to more than 800 m to forage^53,54^. The two pilot whale species, short- and long-finned pilot whales (*G. macrorhynchus* and *G. melas*, respectively), are very difficult to distinguish at sea and as a result species-specific observations in the NEUS, where the two species overlap, are limited and the range of long-finned pilot whales is not well understood ^49^. Observations from stranding events and fisheries bycatch can thus provide important information on the distribution of pilot whales at the species level. Long-finned pilot whales frequently strand in groups of up to several hundred individuals, although the causes of these mass stranding events are not well understood^77^. Strandings enable close examinations of morphology, allowing species-specific trends to be examined for these species which are otherwise difficult to identify. Data on long-finned pilot whale diet in the NEUS are limited, but stomach content analyses found that long-finned squid, short-finned squid, Atlantic mackerel, and Atlantic herring dominated the diet^50^. These four species were therefore used to represent pilot whale prey in our analyses.

Atlantic herring and Atlantic mackerel are two of the most common pelagic fish species in the NEUS with the distribution of Atlantic herring being more northerly than Atlantic mackerel. Both species move south in the winter and undergo an extensive poleward migration in the spring. Atlantic herring are known to undergo large migrations on the order of 1500 km each year^51^. Shifts in the center of biomass have been observed in both of these species that are tightly coupled with sea surface temperature concomitant with increases in population size and area occupied^2,78^.

Long-finned squid and short-finned squid are prey for many fish predators in the Northwest Atlantic and both support commercial fisheries. They are widely distributed with the range of long-finned squid extending from Newfoundland to Venezuela and the range of short-finned squid extending from Iceland to the east coast of Florida^79^. They are annual species and spawn year-round. Like many species in this temperate marine system, long- and short-finned squid migrate offshore and south in the autumn to overwinter and then migrate inshore and north as waters warm in the spring, but their annual migration is much less extensive than Atlantic herring or Atlantic mackerel. These species are known to undertake diel vertical migration and as such must respond to environmental factors throughout the water column^80^. Shifts in distribution over multidecadal time scales have not been detected in these species^81^.

### Data sources

We use data from strandings and fisheries bycatch to assess changes in the distribution of long-finned pilot whales in the NEUS in relation to changes in sea surface temperature (SST) between 1992 and 2016. Strandings data were obtained from the U.S. National Stranding Database collected by the Greater Atlantic Region Marine Mammal Stranding Network (GARMMSN) and the Southeast Marine Mammal Stranding Network (SEMMSN) from 1992 to 2016, which together cover strandings throughout the eastern seaboard of the U.S. Following amendments to the U.S. Marine Mammal Protection Act (MMPA) in 1992, the Marine Mammal Health and Stranding Response Program was formalized resulting in standardized data collection by stranding networks across the country which we used to assess changes in the latitude of strandings through time. Level A data collected by stranding networks in the U.S. must be reported to the National Stranding Database, and includes information such as species identification, date and location of strandings, and the condition of the stranded animal (https://www.fisheries.noaa.gov/national/marine-life-distress/national-stranding-database-public-access). Since the number of pilot whales stranding was not expected to be representative of pilot whales occurring offshore at that time and location, individual pilot whales that stranded on the same day within a distance of 30 km were considered part of the same stranding event when analyzing the latitude of stranding events in each year so as not to bias analyses by giving more weight to larger stranding events. In these cases, the mean latitude of stranding locations of multiple individual was used to represent the latitude of the stranding event. Cape Hatteras has historically been considered to be the southern extent of the range of long-finned pilot whale range^49^, and the strandings data agreed with this assessment. While several strandings of long-finned pilot whales occurred at or just north of Cape Hatteras, only three stranding events were recorded south of Cape Hatteras during the study period (in 1996, 1998 and 2003). These strandings were clear outliers in the dataset, occurring more than 550 km south of other strandings, and were excluded from analyses.

Long-finned pilot whales are taken as incidental catch within the Northeast bottom trawl fishery, which constitutes the majority of annual human-caused mortality of long-finned pilot whales in the NEUS ^49^. We obtained data on bycatch of long-finned pilot whales in the northeast bottom trawl fishery from the Northeast Fisheries Observer Program (NEFOP; https://www.fisheries.noaa.gov/new-england-mid-atlantic/fisheries-observers/northeast-fisheries-observer-program) and the At Sea Monitoring (ASM) Program (https://www.fisheries.noaa.gov/new-england-mid-atlantic/fisheries-observers/sea-monitoring-northeast) run by the National Marine Fisheries Service (NMFS) Northeast Fisheries Science Center (NEFSC). These programs place independent observers aboard bottom trawls to record bycatch and detailed data on each trawl (e.g., haul locations, gear type, target species and catch, incidental bycatch). We used NEFOP data from 1992 on and ASM data from 2010, when the ASM program was initiated, to examine locations and rates of pilot whale bycatch. We refer to these data collectively as observer data. Most occurrences of pilot whale bycatch in observer data (93%) were not identified to species. As noted in stock assessment reports for long-finned pilot whales ^49^, all pilot whale bycatch in bottom trawls occurs in regions is attributed to long-finned pilot whales due to the location of these trawls relative to the typical habitat of long- and short-finned pilot whales. Our analysis of NEFOP and ASM data agreed with this assessment; any pilot whale bycatch identified to the species level consisted exclusively of long-finned pilot whales, and morphological measurements and habitat assessments indicated that observed pilot whales were long-finned pilot whales. For example, all adult female pilot whales that were taken as bycatch were greater than 400 cm in length, and a recent study of pilot whale morphometrics from the eastern seaboard of the United States indicated that mature female short-finned pilot whales are considerably less than 400 cm in length (mean 358.48 ± 4.26 cm for mature female short-finned pilot whales and 432.81 ± 5.82 cm for mature female long-finned pilot whales, respectively^32^. Further, stock assessment reports delineate short- and long-finned pilot whales based on sea surface temperature, with the probability of a pilot whale being a long-finned pilot whale of near 1 at water temperatures < 22 °C ^49^; see also results of this study), and all observations of pilot whale bycatch occurred in temperatures below 22 °C.

Data on the abundance and distribution of longfin squid, shortfin squid, Atlantic mackerel, and Atlantic herring were obtained from the Northeast Fisheries Science Center (NEFSC) fall trawl survey, which uses a stratified random design and has occurred in the spring since 1968 and fall since 1963 (https://www.nafo.int/Portals/0/PDFs/sc/2014/scr14-024.pdf ). Details of the data collection for this survey are outlined in ^82^. Since the 1980s, there has been no significant change in the timing of the fall survey, with surveys conducted between September and December of each year^81^, and the mean annual latitude and longitude of the stations has not changed over time. We used only strata that were consistently sampled (01010-01300, 01360-01400, 01610-01760) and data have been corrected for changes in gear and vessels as outlined in ^2,81^. The NEFSC also conducts a spring trawl survey using the same methodology, but our analyses focused on the fall survey as observations of fisheries bycatch suggested that long-finned pilot whales were most frequently observed in the NEUS between September and February (Supplementary Fig. 9).

We assessed Sea Surface Temperature (SST) in the NEUS using data from the Canadian Meteorological Center (CMC; CMC0.2deg-CMC-L4-GLOB-v2.0; https://podaac.jpl.nasa.gov/dataset/CMC0.2deg-CMC-L4-GLOB-v2.0. ). To match the timing of biological datasets, we used fall (September to November) climatologies of SST to examine changes in SST from 1992 to 2016 within the NEUS and subregions therein (Mid-Atlantic Bight, Southern New England, Georges Bank and Gulf of Maine; Fig. 1A).

### Analyses

We focus analyses on the 1992–2016 time period when we had available data from all biological datasets, and examine poleward distributional shifts in all species by quantifying along-shelf changes in the annual biomass-weighted mean along-shelf location as in previous studies^2,81^. Along-shelf locations were calculated as distances from Cape Hatteras, North Carolina rather than latitudinal changes since changes in latitude do not fully capture along-shelf distances along the curvilinear shelf of the NEUS^2^. Along-shelf distances were calculated using the Spatial Analyst package in ArcGIS (version 10.8.1), while all other analyses were conducted in R (version 4.0.3). We used linear regressions to examine changes in the following parameters over time: mean SST in sub-regions of the NEUS (Fig. 1A), mean along-shelf distance of pilot whale observations from strandings and bycatch, and both biomass-weighted mean along-shelf distance and biomass-weighted mean depth of Atlantic herring, Atlantic mackerel, longfin squid and shortfin squid where depth refers to the bottom depth at that station (i.e., the mean represents depths at which the trawls were conducted, not necessarily the mean depth of all fish of that species in the water column). A poleward shift in the distribution of long-finned pilot whales was evident in both strandings and bycatch data (Supplementary Fig. 10), and we used the mean value of all pilot whale observations (including both strandings and bycatch data) in each year in the linear regression estimating changes in pilot whale distribution through time. We also assessed relationships between the mean along-shelf distance of study species (mean values for pilot whale observations and biomass-weighted along-shelf distances for fish and squid study species) and SST within the core ranges of each species using linear regressions. For fish and squid species, core ranges used for SST and climate velocity analyses were calculated as the 50% Kernel Density Estimate (KDE) within the bottom trawl survey area using the ks package in R. Pilot whale core ranges for SST and climate velocity analyses were calculated differently as strandings are typically recorded on land and therefore locations of strandings do not reflect at-sea habitat use of pilot whales. Thus, we were limited to using bycatch data to define the core habitat of pilot whales for oceanographic analyses. Due to the small sample size of bycatch data relative to available prey data, we defined the bounds of pilot whale habitat using the 25% and 75% quantiles of along-shelf distances and depth values from bycatch data, respectively, rather than using the 50% KDE. In addition, we examined changes in the rate of pilot whale bycatch through time using Bycatch Per Unit Effort (BPUE), defined as the number of trawls in which pilot whale bycatch was observed divided by the total number of trawls observed. Before conducting analyses of pilot whale bycatch, we examined the spatial distribution of observed bottom trawls through time and found no evident temporal bias in observed trawls that would bias analyses of pilot whale bycatch through time (Supplementary Figs. 1, 2). We examined changes in the latitude and SST of trawls and pilot whale bycatch by examining the abundance of trawls and the rate of bycatch within 150 km along-shelf distance bins during each year of analysis. We visualized spatial shifts in pilot whale bycatch through time by calculating rates of BPUE within four bands evenly distributed between the minimum and maximum along-shelf distances at which pilot whale bycatch was observed.

In order to compare our results from 1992 to 2016 period when pilot whale data were available with trends observed for fish and squid over a longer time period, we also performed linear regressions of mean biomass-weighted along-shelf distance and biomass-weighted depth, respectively, vs. year for Atlantic herring, Atlantic mackerel, longfin squid and shortfin squid from 1980 to 2019. This allowed us to assess whether recent rapid warming in the NEUS impacted the relationships observed in fish models.

To assess whether species were shifting faster or slower than expected based on changes in water temperature, we compared observed species shifts to estimates of local climate velocity^17,18,20,83,84^. Analyses of climate velocity provide advantages over examining rates of change of surface isotherms for ecological studies as specific isotherms are relevant to some species more than others, and spatial movements vary considerably between isotherms. Further, there is considerable variability in how isotherms change in space, and thus measuring changes in isotherms in different locations (e.g., on the inner and outer shelf; see Supplementary Fig. 11) can produce considerably different results. We used the approach of Loarie et al.^17^ to examine a holistic metric of climate velocity representing the instantaneous local velocity along the earth’s surface that is needed to maintain constant temperatures. This methodology makes no assumptions about thermal tolerances of individual species, is independent from species distribution data, and does not require specific temperatures or isotherms to be selected, thus allowing climate velocity to be compared in the core ranges of different species. Climate velocity is calculated as the ratio of temporal to spatial temperature gradients (℃/ year and ℃/km, respectively), resulting in an index of the velocity of temperature change (km/year). As with other SST analyses, we used mean fall SST from the CMC at a 0.2 degree resolution to assess temperature gradients, using the slope of a linear model fit through each year of the study period to compute temperature gradients^17^ (Fig. 2). We used the VoCC package^85^ to calculate the spatial gradient in SST in ℃ /km in a 3 × 3 cell neighborhood as in Burrows et al. (2011).

We examined observed distributional shifts relative to climate velocity within the core ranges of each species and examined spatial patterns in the pace of climate change within the NEUS by comparing climate velocities within NOAA Ecological Production Units (Gulf of Maine, Georges Bank, Southern New England, Mid-Atlantic Bight as well as the adjacent Scotian Shelf; Fig. 1A).

## Supplementary Information

Supplementary Information.

## Data Availability

All data needed to evaluate the conclusions of this work are present in the paper. Raw data are available on request from the databases described in the Materials and Methods and Supplementary Materials, with the exception of the fisheries observer data which are not publicly available due to confidentiality of fisheries statistics under US law.
