# Supplementary material for: Trait-mediated shifts and climate velocity decouple an endothermic marine predator and its ectothermic prey
Source: Sci Rep. 2021 Sep 16;11:18507. doi: 10.1038/s41598-021-97318-z (PMC8445949; doi:10.1038/s41598-021-97318-z)

**Supplementary Materials**

**Supplementary Table 1:** Number of pilot whale observations by year.

| **Year** | **No. Stranding events** | **No. bycatch observations** | **Total observations** |
| --- | --- | --- | --- |
| 1992 | 18 | 0 | 18 |
| 1993 | 9 | 1 | 10 |
| 1994 | 5 | 0 | 5 |
| 1995 | 3 | 0 | 3 |
| 1996 | 7 | 1 | 8 |
| 1997 | 6 | 1 | 7 |
| 1998 | 7 | 1 | 8 |
| 1999 | 10 | 2 | 12 |
| 2000 | 5 | 1 | 6 |
| 2001 | 9 | 1 | 10 |
| 2002 | 13 | 0 | 13 |
| 2003 | 18 | 1 | 19 |
| 2004 | 10 | 4 | 14 |
| 2005 | 19 | 12 | 31 |
| 2006 | 6 | 3 | 9 |
| 2007 | 10 | 5 | 15 |
| 2008 | 9 | 9 | 18 |
| 2009 | 11 | 7 | 18 |
| 2010 | 2 | 17 | 19 |
| 2011 | 7 | 14 | 21 |
| 2012 | 7 | 15 | 22 |
| 2013 | 7 | 10 | 17 |
| 2014 | 4 | 7 | 11 |
| 2015 | 0 | 0 | 0 |
| 2016 | 2 | 4 | 6 |
| **All years** | **204** | **116** | **320** |


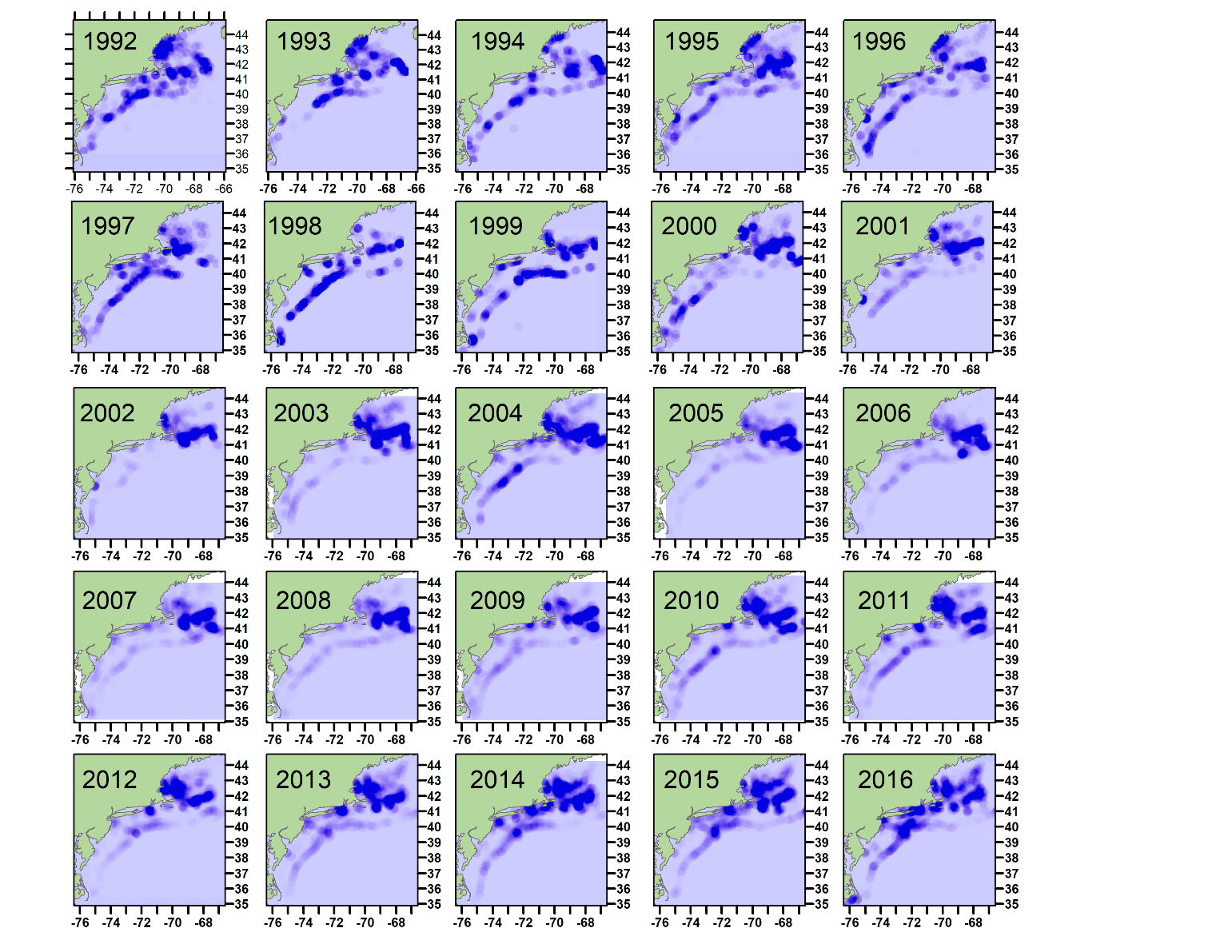
**Supplementary Figure 1**: Spatial distribution of bottom trawls observed by the NEFOP and ASM observer programs by year. Maps produced in ArcGIS (version 10.8.1).

**
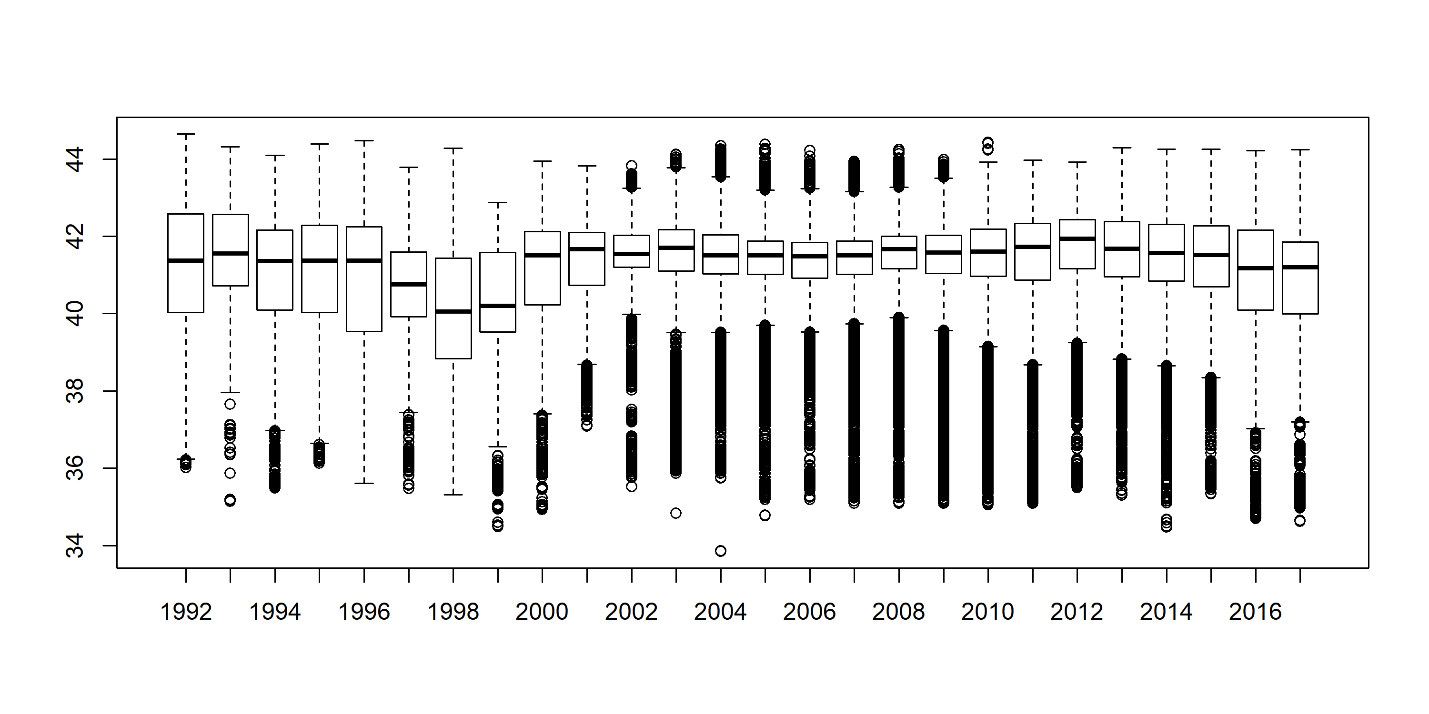
Supplementary Figure 2**: Latitude of bottom trawls observed by the NEFOP and ASM observer programs by year.

**Supplementary Figure 3:** Along-shelf distance of long-finned pilot whale bycatch and bottom trawls observed by the NEFOP and ASM observer programs by year.


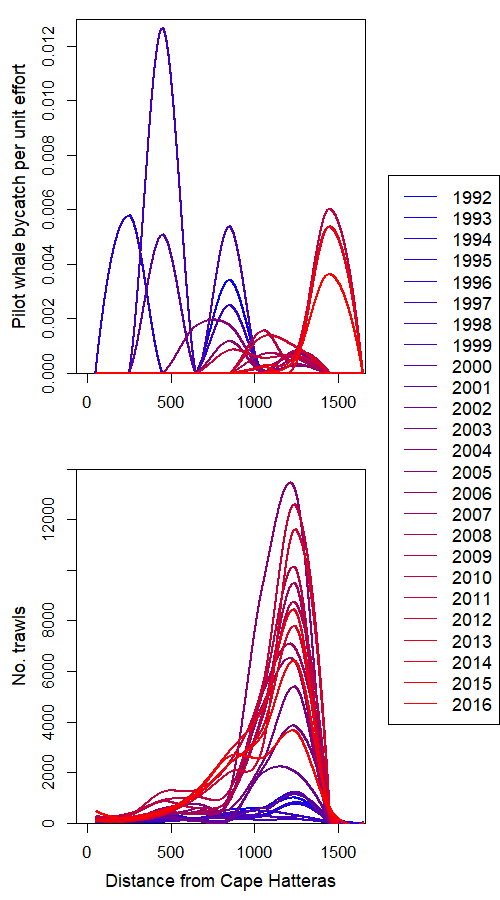


**Supplementary Figure 4**: Annual rates of long-finned pilot whale bycatch per unit effort for bottom trawls occurring below and above 20^o^C, respectively (Wilcoxon test, p = 1.56 x 10^-4^)


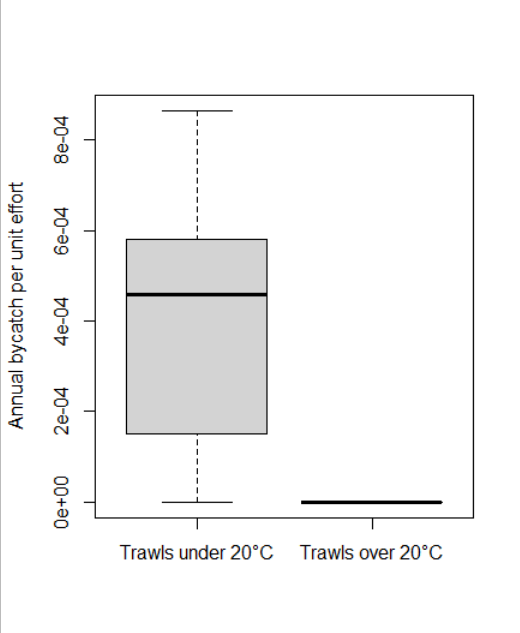


**Supplementary Figure 5**: Spatial distribution of Atlantic mackerel catch in the Northeast Fisheries Science Center fall trawl survey by year. Maps produced in R (version 4.0.3).

**
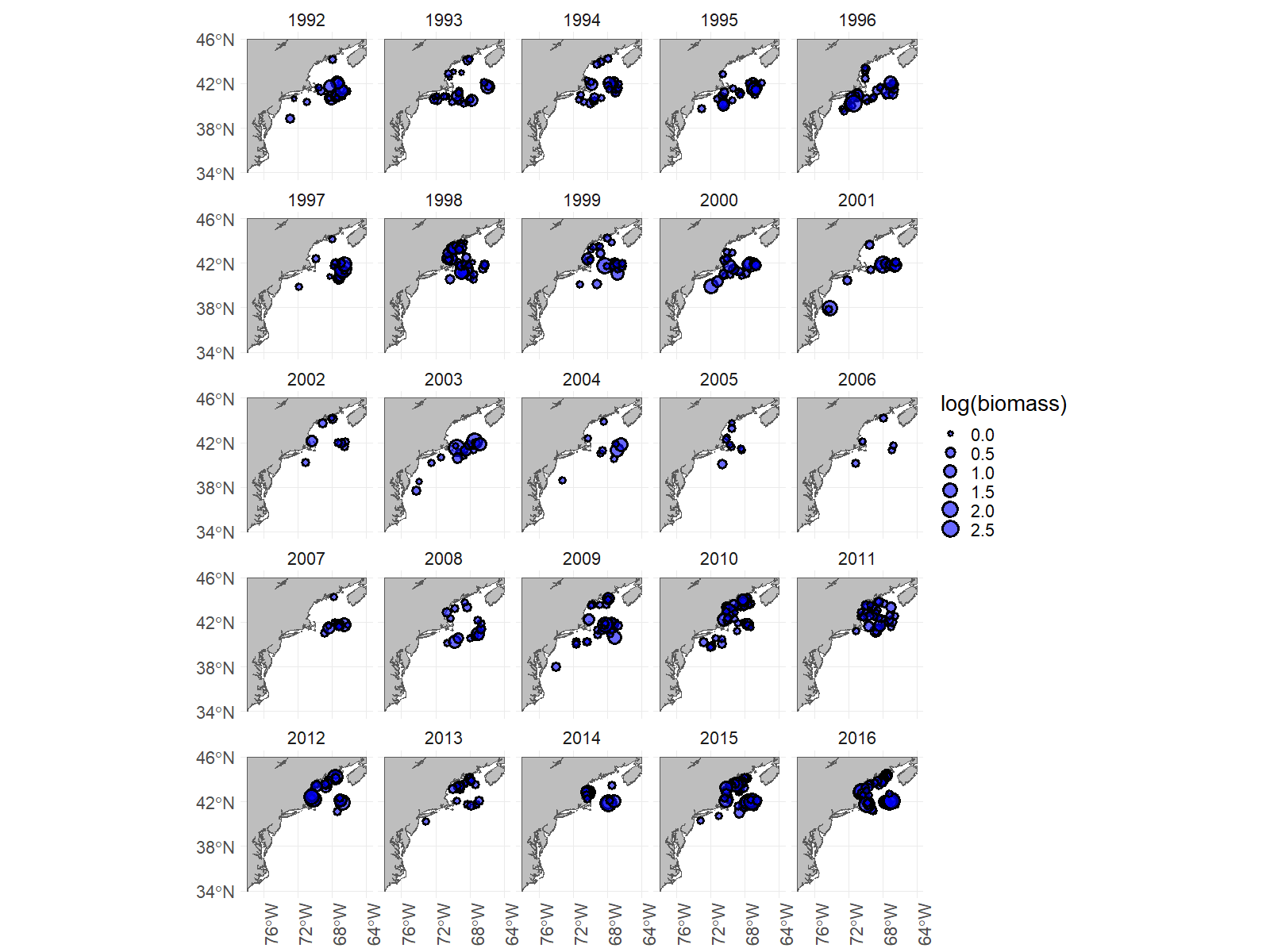
**

**Supplementary Figure 6**: Spatial distribution of Atlantic herring catch in the Northeast Fisheries Science Center fall trawl survey by year. Maps produced in R (version 4.0.3).

**
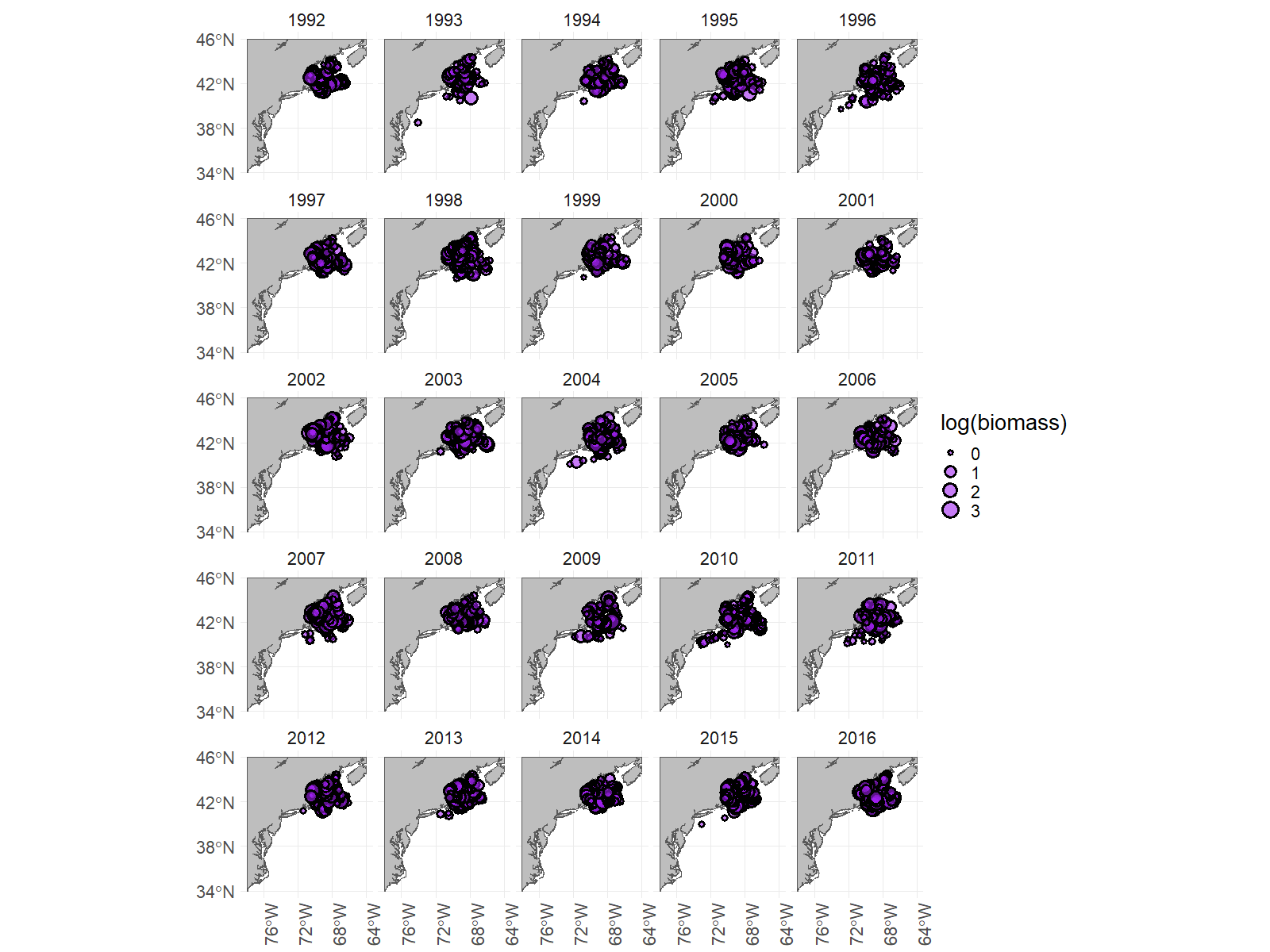
**

**Supplementary Figure 7**: Spatial distribution of longfin squid catch in the Northeast Fisheries Science Center fall trawl survey by year. Maps produced in R (version 4.0.3).

**
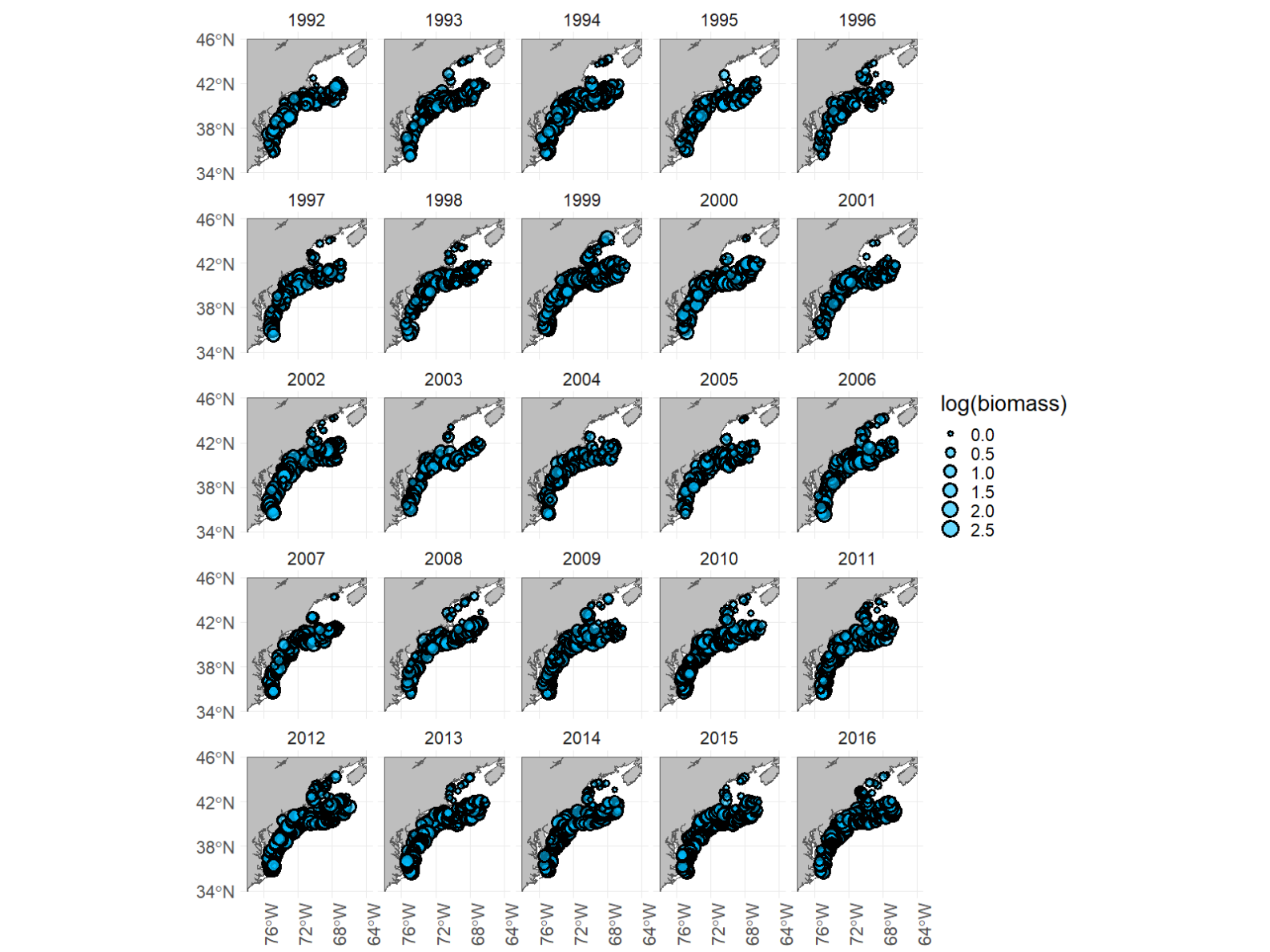
**

**Supplementary Figure 8**: Spatial distribution of short-fin squid catch in the Northeast Fisheries Science Center fall trawl survey by year. Maps produced in R (version 4.0.3).

**
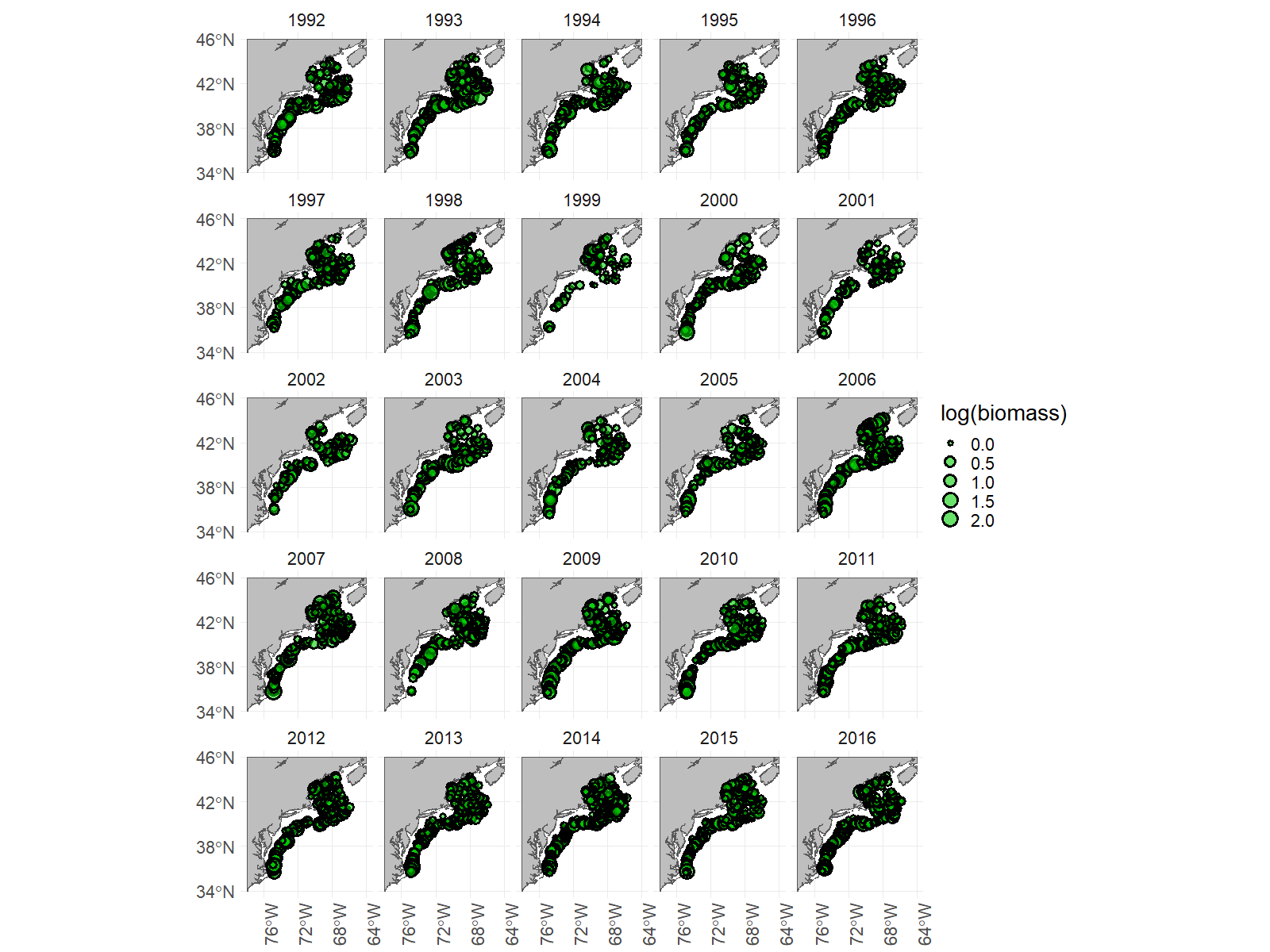
**

**Supplementary Figure 9:** Proportion of years in which pilot whale bycatch was observed in the bottom trawl fishery by month of year


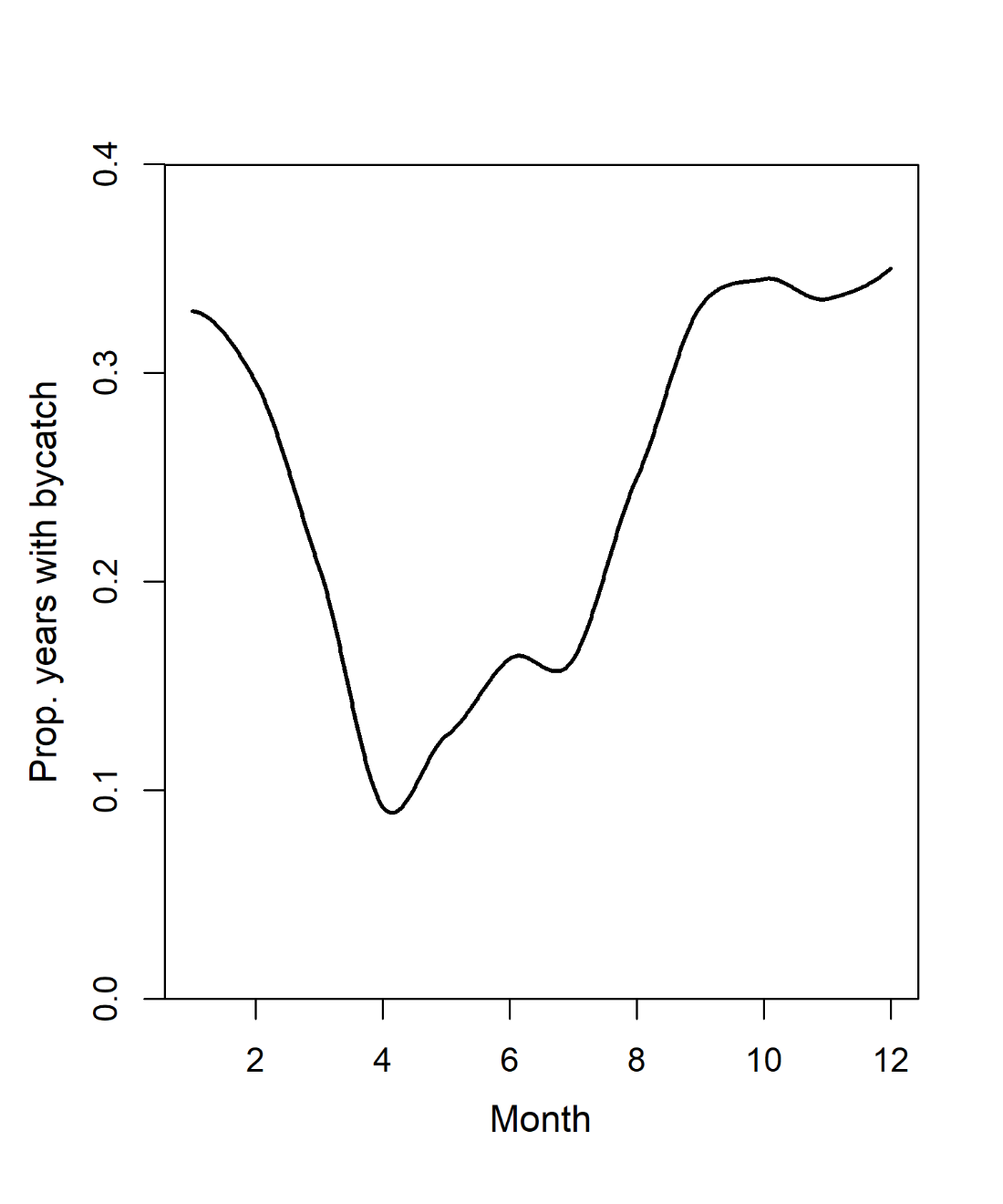


**Supplementary Figure 10**: Mean along-shelf distance for pilot whale observations from strandings (red squares) and bycatch (yellow triangles) datasets, respectively, by year. Analyses used average location by year, incorporating both datasets, to provide the most comprehensive and parsimonious means of presenting this trend. Here we show trends in strandings and bycatch data separately, which demonstrate that both of these two independent datasets show strong poleward shifts in distribution.

**
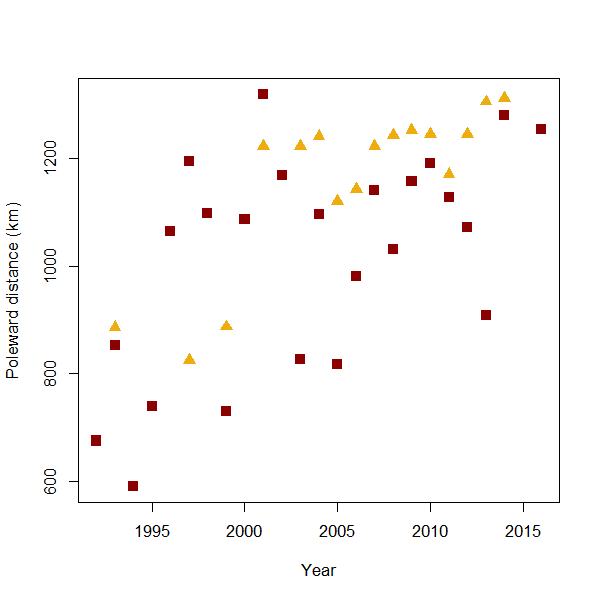
**

**Supplementary Figure 11:** Change in location of the 20ºC isotherm in the Northeast United States calculated for fall (September-November) sea surface temperature from 1992-2016. Map produced in ArcGIS (version 10.8.1).


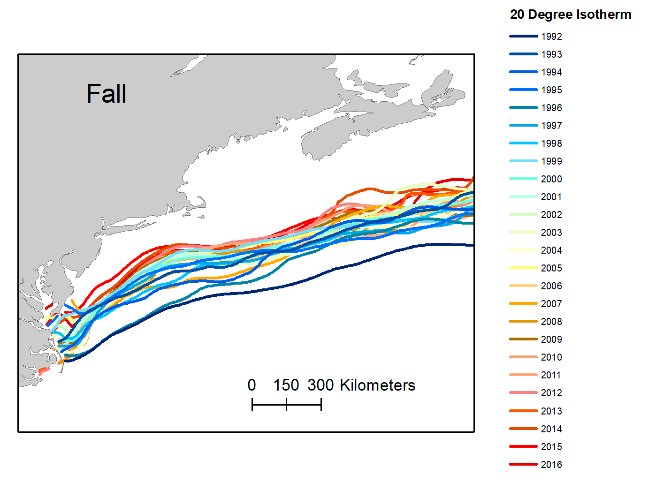

Supplement: Supplementary file 1 — Supplementary Information. [file 41598_2021_97318_MOESM1_ESM.docx]
